# Fifteen-year trends in self-reported racism and link with health and well-being of African Canadian adolescents: a secondary data analysis

**DOI:** 10.1186/s12939-021-01446-x

**Published:** 2021-04-26

**Authors:** Helen U. Okoye, Elizabeth Saewyc

**Affiliations:** University of British Columbia, School of Nursing, Vancouver, British Columbia Canada

**Keywords:** Adolescents, Racial discrimination, Gender, Immigrant, Canadian-born

## Abstract

**Background:**

We assessed the prevalence and trends in racial discrimination among African Canadian adolescents in British Columbia. The association between racial discrimination and self-rated health, access to mental health services, substance use, suicidal thoughts and attempts, experience of extreme stress, among others were examined within the 2018 dataset.

**Methods:**

Secondary analysis used the data collected from African Canadian adolescents (*n* = 2448) as part of the British Columbia Adolescent Health Surveys (2003–2018). We examined whether racial discrimination increased, decreased, or remained stable over time. We evaluated experiences of racial discrimination for all adolescents, and then disaggregated analyses for boys, girls, immigrant, and Canadian-born African adolescents. We used Rao-Scott’s adjusted chi-square to test differences in racial discrimination and adjusted logistic regressions to test trends across survey years, widening or narrowing gaps in racial discrimination, as well as the link to health outcomes.

**Results:**

Racial discrimination was significantly different across the survey years (Adjusted F = 4.60, *p* < .01), with the highest percentage of adolescents reporting past year racial discrimination in 2018 (29.9%) and the lowest percentage in 2013 (21.3%). Girls and immigrant African Canadian adolescents were more likely to have experienced racial discrimination. However, girls and Canadian-born adolescents had the highest odds of reporting racial discrimination in 2018 compared to 2003, AOR = 1.85, and 1.58, respectively. The findings reveal significant differences in the experiences of racial discrimination for boys and girls, as well as for immigrant and Canadian-born African adolescents. Significant differences were noted in the link between racial discrimination and self-rated health and engaging in behaviours that might expose them to health risks. The worst negative health outcomes were found for boys and immigrant African Canadian adolescents.

**Conclusion:**

The study suggests that more than 1 in 4 African Canadian adolescents in British Columbia report racial discrimination, which is an increasing trend in recent years. Those who reported racial discrimination also had the worst adverse health outcomes. There is a need for more public health action to reduce racism, create awareness about the negative health impacts, and provide better support for African Canadian adolescents.

## Background

Racial discrimination is a public health challenge, a social determinant of health, and a major contributor to health inequities among ethnic minority groups [19, 27, 41]. It is the unfair, negative, and prejudiced attitudes and beliefs towards population groups because of their racial identity [60]. Perceived racial discrimination has been consistently linked with stress and disparities in health and illness experiences [5, 36, 54]. For instance, psychological, physiological, and cognitive reactions to experiences of racial discrimination are precursors of mental and physical health problems among individuals who perceive such discrimination [15, 19, 58]. Furthermore, perceptions of racial/ethnic discrimination among racial minority groups is associated with general self-reported poor health, as well as socioemotional distress resulting in substances use, sexual intercourse without the use of adequate protective tools, suicidal thoughts and attempts [33, 59]. However, available evidence indicate that Blacks report more racial discrimination compared to other ethnic minority groups [30, 60].

During adolescence, attitudes and behaviours that influence adult life are formed and become embedded in an individual [6, 54, 55]. Additionally, the developing adolescent brains are still learning to adapt and cope with the socioemotional challenges in the social environment, including experiences of racial discrimination [26]. Thus, at this stage of growth, young people become highly susceptible to the negative impact of racial discrimination as they form their cultural, ethnic, and racial identities. Such racial discrimination on African adolescents living amidst Caucasian-majority groups and other groups contribute to psychological stress, and affect their mental health and well-being [34, 60]. Studies have repeatedly indicated that exposure to racial discrimination in early years, particularly during adolescence, are significant predictors of depressive symptoms in later years among African American adolescents [3, 15, 26].

Recent systematic and meta reviews reveal that most of the studies that examine the experiences of racial discrimination among the Black population are concentrated in the United States, U.S [35, 40]. Also, many studies examine the phenomenon among the adult Black population, while adolescents’ experiences of racial discrimination and the associated health outcomes have received less attention [5, 12, 45]. A large body of evidence in the U.S. has linked experiences of racial discrimination among adolescents to psychological distress, depressive symptoms, substance use, and other behaviours that might expose them to health risks [3, 5, 15]. Gender has been shown to moderate the relationship between racial discrimination and health behaviours among adolescents [8, 42] Not only do experiences of racial discrimination have immediate adverse health outcomes, they have been shown to predict psychological and emotional well-being later in life particularly among men and boys [3]. Studies have documented differential experiences of racial discrimination and associated health outcomes for boy and girls [3, 5, 8, 20, 44]. For example, evidence reveals that although both boys and girls experience adverse mental health outcomes of racial discrimination, these effects lead to long-term psychological deterioration in adult life for adolescent boys than girls [3]. Furthermore, a longitudinal data analysis revealed that cumulative racial discrimination predicts higher use of substances among boys in their young adulthood than for girls [8].

Besides gender differences in the relationships between racial discrimination and health outcomes, many studies have examined this link for first- and second-generation immigrants, albeit, confounding results [17, 52]. For instance, experiences of racial discrimination, and the associated poorer health outcomes are higher among first generation immigrants, [52], particularly during adolescence [16, 39]. Even more so as acculturation and language barrier may worsen these experiences and lead to increase in experiences of stress, as shown among first-generation immigrant adolescents in the U.S. [48]. Additionally, the healthy immigrant effect explains the better health outcomes that have been documented among people who move from developing to developed countries [16, 25].. Evidence suggests that first generation immigrants are healthier than their native-born counterparts, given the selective immigration policies in the destination country. Also, the higher social support from the African community in the recipient country might explain the differential health outcomes [7, 16, 25, 52]. However, the immigration-health paradox tends to fade off with longer years in the host country [16, 47]. Given that majority of these studies are in the United states, evidence is still scant about the relationship between experiences of racial discrimination and the well-being of first-generation immigrant and Canadian-born African adolescents.

The Black population has only increased in British Columbia in recent years and extrapolating findings from the U.S. to Canada may be inaccurate [56]. Black Canadians represent 3.5% of Canada’s population and accounts for 15.6% of the minority ethnic groups in Canada [50]. Further, British Columbia is the most culturally diverse province in Canada, and the third largest province in the country, but the province hosts a very small number of Black Canadians. The ethnic group represents 1% of the Province’s population [50]. Thus making them a visible minority ethnic group in the province [50].

As the Canadian Government commits to promoting mental health and well-being across the life span and reducing health disparities, there is a need to investigate the experiences of racial discrimination among Black Canadian adolescents, and the changes in experiences of racial discrimination over time. A limited number of studies have investigated experiences of racial discrimination among Black Canadian adults and college students in Canada [24, 53, 56]. However, the prevalence and trends in racial discrimination among African Canadian adolescents in British Columbia have received less attention, despite relevant associations of racial discrimination with adverse health outcomes among individuals in this age group.

The current study sought to fill this critical gap in Canadian data, by assessing the prevalence and trends in racial discrimination among African Canadian adolescents in British Columbia. The study evaluated the links between racial discrimination and well-being of African Canadian adolescents. Also, experiences of racial discrimination and the link with health outcomes were assessed separately for boys, girls, first generation immigrant African adolescents and African adolescents born in Canada.

### Purpose of the study

The study examined the prevalence and 15-year trends in racial discrimination, as well as the link between experiences of racial discrimination and health outcomes using data obtained from the province-wide British Columbia Adolescent Health Survey (BCAHS). The McCreary Centre Society (MCS) conducts the BCAHS every five years among a representative sample of students in Grades 7 to12. The survey includes questions about adolescents’ ethnic background and experiences of discrimination related to race, ethnicity, or skin colour, in addition to other questions that address adolescents’ health behaviour.

### Research questions

The current study addressed four questions relating to racial discrimination among African Canadian adolescents: (1) What is the prevalence of past year racial discrimination among African Canadian Adolescents in British Columbia in the relevant survey years? (2) Was there a significant difference in experiences of racial discrimination for boys versus girls, and for immigrant versus Canadian-born African adolescents? (3) Was there a significant difference in trends (decreased, increased, or unchanged) of racial discrimination among the adolescents between 2003 and 2018? (4) Was there a link between experiences of racial discrimination and self-rated health, experience of extreme stress, access to health services, substance use, suicidal thoughts and attempts among African Canadian adolescents in British Columbia?

## Method

### Survey design and procedure

The BCAHS is a cross-sectional school-based survey that is conducted every five years using cluster-stratified sampling to select provincially representative students in classrooms. The survey examines risks and protective factors that influence adolescent health behaviours, including exposure to violence and discrimination as well as youth assets, family, school and community connectedness [43, 49]. The sampling frame used for the surveys was comprised of enrolled students who were in grades 7 to 12 (ages 12 to 18 years) in public school districts in British Columbia. Samples in each year of the surveys were randomly selected from classrooms stratified by grade and health service delivery area (HSDA) across the province. Data obtained from the surveys are weighted to adjust for the complex survey design and scaled to represent the population of enrolled students. The number of participating school districts have increased steadily since the first survey in 1992, and in 2018, 58 out of the 60 school districts in the province participated in the survey [29, 43]. While the questionnaire has mostly remained the same over the years, allowing for trend analyses, some questions have been updated to reflect current social issues [43, 46]. The paper and pencil questionnaire of 140-items is usually administered by public health nurses and nursing students and completed anonymously by the students.

### Sample selection

The last four surveys of the BCAHS from years 2003, 2008, 2013 and 2018 were used for the trend analysis because questions about ethnicity prior to 2003 did not include African Canadian adolescents. The total sample of school students who participated in the BCAHS between 2003 and 2018 was 126,994. The unweighted sample of African Canadian adolescents in the survey years under review was 2448, ranging between 439 and 808 from 2003 to 2018. The weighted, scaled sample size of African Canadians adolescents ranged from 4651 in 2003 to 5744 in 2018, with a total of 21,834 across all 16 years. Given that the survey includes grades 7 through 12, it is possible that some adolescents might have participated in the survey twice, but since the overall sample is only about 10% of enrolled students in each sample year, it is unlikely that students have participated in the survey twice, given the few participants in any year.

### Inclusion and exclusion criteria

For the current study, participants were included in the analysis if they (1) self-identified as African (2) responded to the relevant survey questions in the relevant survey years from 2003 to 2018. To ensure reliable trends, only school districts that participated in at least three of the four survey years (94.2%) were included in this analysis. Also, adolescents who did not indicate their gender (0.1%) were excluded from the analysis.

### Measures

The dependent variable for this analysis was experiences of racial discrimination in the past year, based on the question, “In the past 12 months, have you been discriminated against or treated unfairly because of your race, ethnicity or skin colour?” Response options were yes or no. Several additional variables from the BCAHS were used to assess the link between racial discrimination and health and well-being of African Canadian adolescents: being sexually active or having given or received oral sex, ever smoked a cigarette, used alcohol, or other substances (e.g., cocaine, heroin, amphetamine, crystal methamphetamine, hallucinogens), as well as self-rated health, and access to mental health and emotional health services. Variables were originally coded or recoded as binary (yes or no). The socio-demographic variables included in the analysis were age, gender, and birthplace. The participant’s birthplace was a dichotomous variable that measured first-generation immigrants, subsequently referred to as immigrants, or Canadian-born adolescents. Gender was measured as a binary variable (boys and girls) in the survey years prior to 2018, but 2018 included both the sex assigned at birth and a gender identity question that offered more than binary options. To provide a consistent measure of gender for reliable trends across years, gender in this analysis was based on sex assigned at birth.

### Data analysis

All analyses were performed with the Complex Samples analytical procedures incorporated in IBM SPSS 26 [22]. Complex Samples procedures not only adjust for unequal probabilities of selection that are associated with clustering and stratification, but also the use of weights. The alpha criterion was set at 0.05 for all analyses. Descriptive statistics, including percentages, means and standard deviations assessed the age distribution of respondents. The SPSS Complex Samples General Linear Models assessed significant differences in age distribution of the respondents across the survey years. The prevalence of racial discrimination was evaluated for each survey year for all respondents, and then disaggregated by gender and birthplace (immigrant vs. Canadian-born African adolescents). Rao-Scott’s chi-square statistic was used to test the differences in prevalence of racial discrimination across the survey years to account for the clustered data.

To determine whether there were statistically significant different trends in racial discrimination over time, logistic regression analyses were conducted to compare data for 2003, 2008, 2013 and 2018 survey years, with age as a covariate to control for potential differences in sample distributions across years. The logistic regression model assessed whether racial discrimination had increased, decreased, or remained stable in 2008, 2013 and 2018, compared to 2003. Age-adjusted odds ratios (AOR) were used to interpret the result of the analyses, where significant AOR greater than or less than 1 shows increasing or decreasing trends, respectively.

Furthermore, for any observed differences of experiences of racial discrimination between boys and girls, and immigrant versus Canadian-born African Canadian adolescents, we tested whether such differences widened, narrowed, or remained unchanged between groups across the given years. We used a recent approach for examining trends and identifying trends in the gaps between groups developed by Homma, Saewyc, and Zumbo [21]. Interaction terms by gender and survey year were included in the regressions, with girls and 2003 survey year as the referent groups, as well as interactions by birthplace and survey year, with Canadian-born adolescents and 2003 survey year as referent categories. The interaction term is a ratio of odds ratios, which compares the AOR of racial discrimination by adolescent’s gender or birthplace for the specified year (e.g., 2018) to the AOR of racial discrimination by gender or birthplace for the referent year (e.g., 2003). Significant interaction odds would indicate that change over time was different for boys and girls, or for Canadian-born versus immigrant African adolescents. This shows that gaps in experiences of racial discrimination for boys, girls, immigrant, or Canadian-born adolescents may be narrowing or widening. The interpretation of the odds ration (OR), and interaction terms are shown in Table 1. The main OR and interaction OR were examined to determine whether the gap widened or narrowed, as suggested by Homma et al. [21]. We controlled for age of respondents in all logistic regression models.

**Table 1:** Interpretation of Odds Ratio for Gap Analysis

	Original ORs	ORs for interaction terms	Racial discrimination
Year 2008, 2013 or 2018	> 1	> 1	Widening
Year 2008, 2013 or 2018		< 1	Narrowing
Year 2003 (reference)		< 1	Narrowing
Year 2008, 2013, or 2018	< 1	> 1	Narrowing
Year 2008, 2013, or 2018		< 1	Widening
Year 2003 (reference)		< 1	Widening

Note. Main effect ORs for a specified year and referent year > 1, interaction ORs > 1 = Gap widening. Interaction ORs < 1 = Gap narrowing. Main effect ORs for a specified year and referent year < 1, and Interaction ORs > 1 = Gap narrowing. Interaction ORs < 1 = Gap widening. ^a^ ORs; Odds ratios from logistic regression models that examined racial discrimination within 2003, 2008, 2013, 2018 survey years

Finally, using the 2018 data only, bivariate logistic regressions were used to assess the link between experiences of racial discrimination and self-rated health, experiences of stress, access to health services, as well ever smoked a cigarette or used alcohol, among others. The results were interpreted with the adjusted odds ratios and confidence intervals.

Adapted with permission from Homma et al. [21].

## Results

### Socio-demographic profile

Descriptive statistics show that most of the African Canadian adolescents were 18 years old or younger (98.6%). In these survey years, more than half of the population of African Canadian adolescents were boys (52.1 to 52.5%). All the African Canadian adolescents identified as either boys or girls in 2003, 2008, and 2013. In 2018, almost all the African Canadian adolescents (99.9%) indicated their gender as the sex assigned at birth. Disaggregated data for immigrant and Canadian-born African adolescents indicate that a greater percentage (69.5%) were born in Canada.

The mean age of the weighted sample of all adolescents was 15.1 years, and there was no significant difference in the age distribution of all African Canadian adolescents in 2008 (*p* = .25), 2013 (*p* = .28, and 2018 (*p* = .06) compared to 2003. However, boys were significantly younger in 2013 compared to 2003 (*p* < .05). On the other hand, adolescent girls were significantly younger in 2008 and 2018 compared to 2003 (p < .05). While there was no significant age difference in the mean age of Canadian-born adolescents in 2008 (*p* = .16), 2013 (*p* = .22) and (2018, *p* = .56) compared to 2003. The immigrant African Canadian adolescents were significantly younger in other years compared to 2003 (*p* < .01). Further, majority of the African Canadian adolescents (86.7%) had lived in Canada for six years or longer. Only a very small percentage (4.6%) of the adolescents lived in Canada for less than two years. From 2003 to 2018, most of the adolescents (93.2 to 92.3%) lived with their parents, and another 6.1 to 5.2% lived with adults who were not their parents.

### Prevalence of racial discrimination by gender, and for immigrant and Canadian-born adolescents

**Fig. 1:**
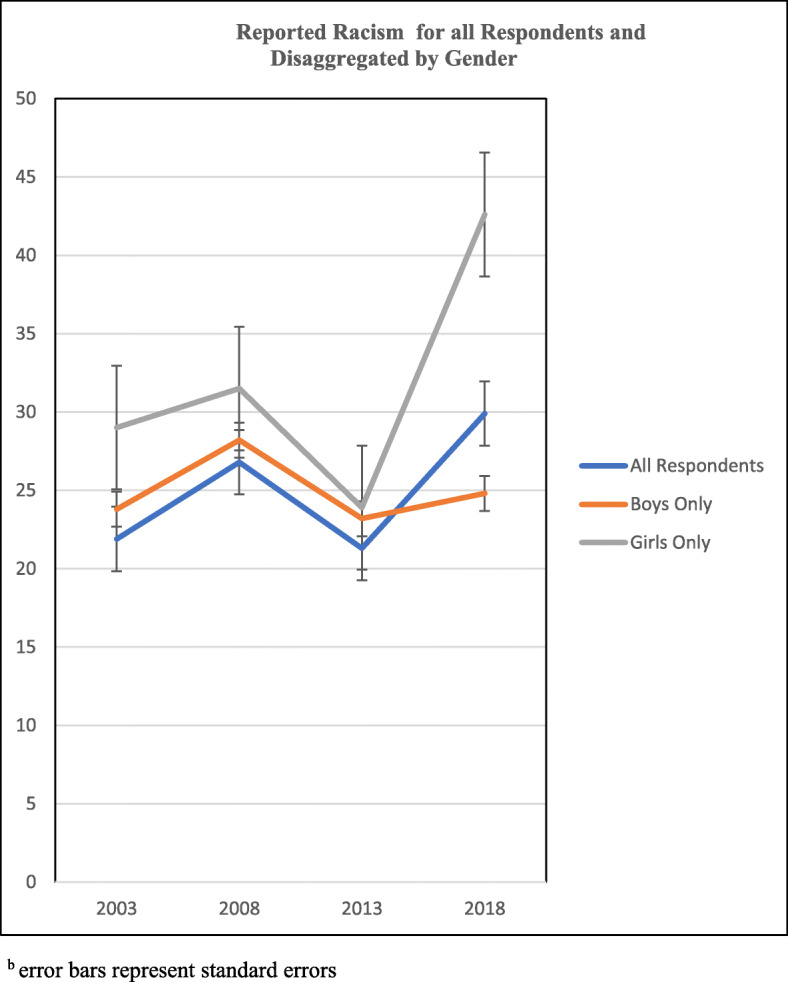
Reported Racism for all Respondents and Disaggregated by Gender

**Fig. 2:**
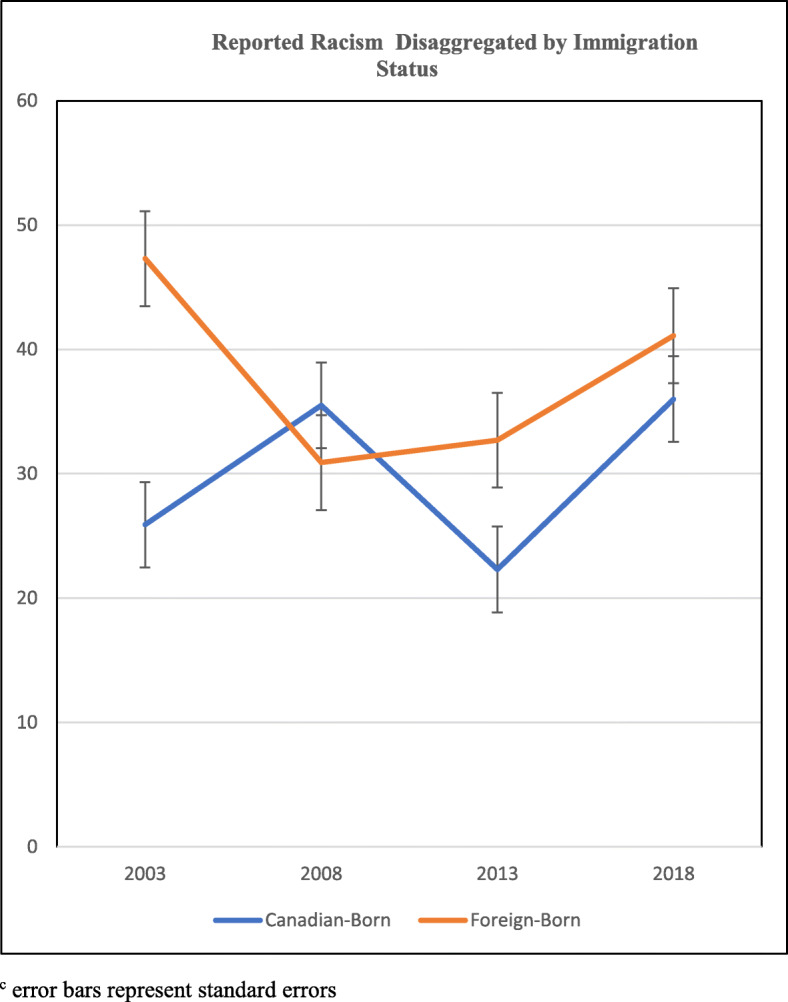
Reported Racism Disaggregated by Immigration Status

Figure 1 depicts the unadjusted 15-year prevalence estimates of racial discrimination for all African Canadian adolescents, and then disaggregated by gender. The results reveal that racial discrimination was highest in 2018 and lowest in 2013. These experiences remained higher for adolescent girls compared to boys in all the years, were lower for both groups in 2013 compared to 2018, but increased exponentially for girls in 2018. The unadjusted 15-year prevalence of racial discrimination disaggregated for immigrant and Canadian-born adolescents is shown in Fig. 2**.** Immigrant African Canadian adolescents reported higher prevalence of racial discrimination in 2003, 2013 and 2018 compared to Canadian-born adolescents. These findings were further examined controlling for age in logistic regression models to determine significant changes over the 15-year period.

### Logistic regression analyses of racial discrimination across survey years

#### Differences for boys, girls, immigrant, and Canadian-born African Canadian adolescents

The age-adjusted logistic regression analyses (Table 2) reveal that there were no significant differences in racial discrimination by gender in 2003, 2008 or 2013. However, in 2018, girls were 59% more likely than boys to have reported racial discrimination *(p < .01).* Compared to Canadian-born adolescents, immigrant African Canadian adolescents were 113% as likely to have reported racial discrimination in 2003 *(p < .05)* and 65% more likely to have reported racial discrimination in 2013 *(p < .05).* Racial discrimination did not significantly differ between Canadian- and immigrants adolescents in either 2008 or 2018.

**Table 2:** Racial Discrimination Stratified by Gender, and for Immigrant and Canadian-born Adolescents

	All Respondents**	Boys Only*	Girls Only**	Boys (vs Girls) ^‡^	Immigrant (vs Canadian-born) ^‡^
	AOR (95% CI)	AOR (95% CI)	AOR (95% CI)	AOR (95% CI)	AOR (95% CI)
2003	21.9 (18.0, 26.4)	23.8 (19.1, 29.2)	29.0 (21.2, 38.2)	1.50 (.91, 2.48)	2.13 (1.13,4.03) *
2008	26.8 (23.8, 30.2)	28.2 (24.4, 32.4)	31.5 (25.6, 38.2)	1.27 (.85, 1.90)	0.81 (0.51,1.28)
2013	21.3 (18.4, 24.5)	23.2 (19.5, 27.3)	23.9 (18.9, 29.8)	1.15 (.79, 1.67)	1.65 (1.08,2.50) *
2018	29.9 (26.9, 33.2)	24.8 (21.2, 28.8)	42.6 (37.4, 47.9)	.63 (.46, .87) **	1.23 (0.85,1.78)

*p < .05, **p < .01, ***p < .001, AOR = Age adjusted odds ratio. ^‡^Referent group: Girls, Canadian-born adolescents

#### Trends in racial discrimination

The trend analyses (Table 3) indicate that the odds of racial discrimination were lowest in 2013 for boys, girls, immigrant, and Canadian-born adolescents. Experiences of racial discrimination were significantly lower for boys in 2013 compared to 2003 and 2008. Adolescent girls had higher odds of reporting racial discrimination in 2018 compared to the previous years. For instance, girls were 85 and 60% more likely to have experienced racial discrimination in 2018 compared to the experiences of racial discrimination in 2003 and 2008, respectively. Also, adolescent girls were 137% more likely to have reported racial discrimination in 2018 compared to 2013. On the other hand, in 2018, Canadian-born adolescents were 58 and 95% more likely to have reported racial discrimination compared to 2003 and 2013, respectively, while immigrant African adolescents had significantly declining prevalence between 2003 and 2013. Also, the increase in prevalence of racial discrimination in 2018 was not significantly different compared to the other years for immigrant adolescents.

**Table 3:** Trends in Racial Discrimination by Year disaggregated by Gender and Birthplace

	Boys Only	Girls Only	Canada-born	Immigrants
	AOR (95% CI)	AOR (95% CI)	AOR (95% CI)	AOR (95% CI)
Model 1				
2008 (vs 2003) ^a^	0.98 (0.62,1.56)	1.15 (0.70,1.91)	1.55 (1.02,2.36) *	0.53 (0.30, 0.94) *
2013 (vs 2003) ^a^	0.60 (0.38,0.96) *	0.78 (0.47,1.29)	0.81 (0.53,1.25)	0.57 (0.33, 0.99) *
2018 (vs 2003) ^a^	0.79 (0.50,1.24)	1.85 (1.17,2.94) **	1.58 (1. 07,2.35) *	0.83 (0.49, 1.41)
Model 2				
2013 (vs 2008) ^b^	0.61 (0.43,0.87) **	0.68 (0.44,1.03)	0.52 (0.37,0.74) ***	1.08 (0.66, 1.76)
2018 (vs 2008) ^b^	0.80 (0.57,1.12)	1.60 (1.12,2.30) **	1.02 (0.76,1.38)	1.56 (0.97,2.49)
Model 3				
2018 (vs 2013) ^c^	1.31 (0.93,1.85)	2.37 (1.64,3.44) ***	1.95 (1.43, 2.65) ***	1.45 (0.93, 2.26)

*p < .05, **p < .01, ***p < .001. Referent Year: ^a^2003, ^b^2008, ^c^2013

#### Gaps in racial discrimination by adolescents’ gender and birthplace

The gap analysis shown in Table 4 reveal that the gap in experiences of racial discrimination between boys and girls remained unchanged in 2008 *(p = 0.60)* and 2013 *(p = 0.41)* compared to 2003, but significantly narrowed in 2018 compared to 2003 *(p < .01).* However, adolescent girls still had the highest odds of reporting racial discrimination in 2018 compared to other years *(p < .01).* The gap between immigrant- and Canadian-born adolescents in experiences of racial discrimination narrowed in 2008 (p < .01) compared to 2003 but remained unchanged in 2013 *(p = 0.33)*, and 2018 *(p = 0.06)*.

**Table 4:** Year by Gender and Year by Birthplace Interactions Effects on Experiences of Racial Discrimination

	AOR (95%CI)
**Model 1**	
Gender Gap	
Boys	1.49 (0.91, 2.45)
2008	1.27 (0.85, 1.89)
2013	1.15 (0.79, 1.67)
2018	1.87 (1.18, 2.97) **
Boys × 2008 (vs 2003) ^a^	0.84 (0.45, 1.59)
Boys × 2013 (vs 2003) ^a^	0.77 (0.42, 1.43)
Boys × 2018 (vs 2003) ^a^	0.42 (0.23, .76) **
**Model 2**	
Birthplace	
Immigrant	2.34 (1.31, 4.17) **
2008	1.55 (1.01, 2.36) *
2013	0.81 (.53, 1.25)
2018	1.58 (1.07, 2.36) *
Immigrant × 2008 (vs 2003) ^a^	0.34 (0.17, 0.72) **
Immigrant × 2013 (vs 2003) ^a^	0.70 (0.35, 1.43)
Immigrant × 2018 (vs 2003) ^a^	0.52 (0.26, 1.03)

*p < .05, ** p < .01, ***p < .001. Referent Year, Gender, and Birthplace: ^a^2003, Girls, Canadian- born adolescents

### Racial discrimination and link with adolescents’ health and wellbeing

The link between racial discrimination and health and wellbeing of African Canadian adolescents were assessed using the 2018 dataset. Bivariate logistic regression models were used to assess the association of racial discrimination with self-rated health, experiences of stress, access to health services, substance use, sexual intercourse without the use of adequate protective tools, among others. The findings reveal that self-rated health was poorer for adolescents who reported racial discrimination, and they had a higher likelihood of engaging in behaviours that could put them at risk for adverse health outcomes compared with those who did not report racial discrimination (Table 5). These adolescents who reported racial discrimination were 48 and 46% more likely to have ever smoked a cigarette or used any substances (e.g., cocaine, heroin, e.t.c.) respectively, compared to those did not report experiencing racial discrimination. Additionally, adolescents who experienced racial discrimination were 97% more likely to have considered suicide and more than two times as likely to have attempted suicide *(p < 0.5)* than adolescents who did not report experiencing racial discrimination. These adolescents also significantly reported extreme stress and not receiving needed emotional and mental health services, 68 and 121% respectively.

**Table 5:** Logistic Regression of Racial Discrimination, Health Behaviours and Access to Health Services

	AOR (95% CI)
Cigarette smoking	1.48 (1.03,2.11) *
Substance use (e.g., heroin, cocaine)	1.46 (1.02,2.09)
Suicidal thoughts	1.97 (1.32,2.94) **
Suicidal attempt	2.05 (1.10,3.83) *
Good Health	0.62 (0.43, 0.89) **
Extreme Stress	1.68 (1. 08,2.61) *
Did not receive mental health services	2.21 (1.54, 3.17) ***

*p < .05, ** p < .01, ***p < .001

Regression Analysis of Racial Discrimination and Health and Well-being Disaggregated by Gender

The results show that experiences of racial discrimination were significantly associated with engagement in behaviours that expose young people to health risks for both boys and girls, but adolescent boys had the worst health outcomes (Table 6). African Canadian boys who experienced racial discrimination were significantly more likely to have ever smoked a cigarette *(p < .05)*, and used other substances, such as cocaine, and heroine *(p < .01)*. The boys also significantly reported general poor health, extreme stress, suicidal attempt, and were more likely to be sexually experienced, e.g., having had sex, given, or received oral sex *(p < .05)*, compared to adolescent girls. While adolescent girls who experienced racial discrimination were two times as likely to have considered suicide compared to those who did not report racial discrimination, boys were three times more likely to have attempted suicide *(p < .05).* Regardless of gender, African Canadian adolescents who experienced racial discrimination were significantly less likely to have received emotional and mental health services *(p < .01)*, compared to those who did not report experiencing racial discrimination.

**Table 6:** Regression Analyses of Racial Discrimination and Health and Well-Being Disaggregated by Gender

	Racial Discrimination	
	Boys AOR (95% CI)	Girls AOR (95% CI)
Ever had a drink of alcohol	1.38 (0.88,2.16)	1.14 (0.72,1.82)
Cigarette smoking	1.73 (1.07,2.79) *	1.26 (0.74,2.15)
Substance use (e.g., heroin, cocaine)	2.15 (1.37,3.37) **	0.96 (0.56,1.64)
Sexually experienced (Given or received oral sex and/or ever had sex)	1.65 (1.03,2.64) *	1.000 (.60,1.66)
Good/Excellent	0.50 (0.29,0.88) *	0.77 (0.48,1.22)
Extreme Stress	2.52 (1.21,5.26) *	1.24 (0.73,2.08)
Have considered suicide	1.53 (0.83,2.83)	2.01 (1.20,3.34) **
Attempted suicide	2.94 (1.09,7.91) *	1.61 (0.72,3.60)
Did not receive emotional and mental health services	2.24 (1.13,4.42) *	1.93 (1.24,3.01) **

*p < .05, ** p < .01

Racial Discrimination and Association with Health and Well-Being Disaggregated by Birthplace

The differences in the experiences of racial discrimination and the link with adolescents’ health and well-being are shown in Table 7. The results indicate that Canadian-born African adolescents who reported racial discrimination were significantly more likely to report poor health outcomes. For instance, 61% reported general poor health, 91% reported extreme stress while 97% said that they did not receive needed emotional and mental health. However, immigrant African Canadian adolescents who experienced racial discrimination by far engaged in coping strategies that may affect health and well-being. They were more than two times as likely to have ever smoked cigarette or used substances *(p < .01)*, five times more likely to have considered suicide *(p < .001)*, four times more likely to have attempted suicide *(p < .05)*, and more than 3 times as likely to report not receiving needed emotional and mental health services *(p < .001).*

**Table 7:** Logistic Regression of Racial Discrimination and Health Outcomes Stratified by Birthplace

	Racial Discrimination	
	Canadian-Born AOR (95% CI)	Immigrants AOR (95% CI)
Cigarette smoking	1.02 (0.63,1.67)	2.73 (1.51,4.95) **
Substance use (e.g., heroin, cocaine)	1.08 (0.70,1.68)	2.55 (1.37,4.75]) **
Good health	0.62 (0.40,0.95) *	0.59 (0.32,1.09)
Extreme Stress	1.91 (1.21,3.28) **	1.07 (0.43,2.66)
Considered suicide	1.54 (0.98,2.42)	4.66 (2.26,9.58) ***
Attempted Suicide	1.64 (.80,3.36)	3.94 (1.15,13.55) *
Did not receive mental health services	1.97 (1.30,3.01) **	3.60 (1.80,7.20) ***

*p < .05, ** p < .01, ***p < .001

## Discussion

This study examined fifteen-year trends in perceived racial discrimination and the link with health and well-being among African Canadian adolescents in British Columbia Canada. The findings of the study are consistent with reports of racial discrimination among adolescents and other minority ethnic groups, including African American adolescents in the United States and elsewhere [5, 19, 35]. The findings reveal an increasing trend in racial discrimination in 2018 after declines between 2003 and 2013, which is troubling, and may be connected with the political changes and the increased visibility of White supremacy and anti-immigrant sentiments in North America [1].

The gender differences in experiences of racial discrimination found in this study are similar to other studies in the U.S. which found that experiences of racial discrimination are different for African American boys and girls [3]. African Canadian adolescent girls reported greater prevalence of racial discrimination than boys in this study, which might be explained by girls demonstrating an awareness of the lower social status of women, hence might develop more sensitivity to discriminatory treatment [9]. In contrast to our findings, other studies found higher levels of racial discrimination among African American boys [10, 23]. However, Cogburn, Chavous, & Griffin [11] found no gender differences in racial discrimination, but boys reported more gender-based discrimination. The findings of this study suggest that, in different locations, racial discrimination might intersect with other forms of discrimination, such as sexism.

Our results also show that racial discrimination was reported by both immigrant and Canadian-born African adolescents, but immigrant adolescents reported higher levels of racial discrimination for most years. Experiences of racial discrimination by immigrant adolescents corroborates other studies that have documented a higher level of racial discrimination experiences among immigrant minority groups [19]. Canadian-born adolescents may be better acculturated than the immigrant African adolescents, which explains the differences in racial discrimination between immigrant and Canadian-born African adolescents [4, 14]. On the other hand, lower proficiency in the English Language and darker skin colour were associated with higher levels of racial discrimination [2]. However, the increase in racial discrimination in the past five years, even for Canadian-born adolescents, suggests a growing trend in overt racism, considering the racial sentiments in recent years across Europe and North America. For instance, researchers have documented the adverse effects of anti-immigration policies on the health and well-being of immigrants and refugees in the United States [1, 37].

Our study, not surprisingly, corroborated that racial discrimination is also associated with several adverse health outcomes among African Canadian adolescents. Those who experienced racial discrimination had a higher likelihood of reporting poorer health, psycho-emotional health issues, not receiving needed emotional and mental health support. These adolescents also engaged in behaviours that can negatively affect their health and well-being compared to those who did not report racial discrimination. These results match the findings of other studies that have documented that experiences of racial discrimination among adolescents are associated with depressive and internalizing symptoms, externalizing behaviours, condom less sex, and having had multiple sexual partners [5, 12, 35, 36, 45, 51]. Hence, there is a need to address not only interpersonal racism, but also all forms of systemic racism and barriers that continue to jeopardize the health and well-being of Black Canadian adolescents, and other racialized groups in Canada [18, 38]. Governmental and non-governmental actions are needed to promote antiracist laws, regulations, and policies as well as attitudinal change at multiple levels of the society that will reduce the social and health inequities experienced by Black Canadian adolescents.

Furthermore, the greater use of substances among those who reported racial discrimination, necessitates that approaches for reducing harms that are associated with drug use consider the social circumstances of people who use drugs. A health equity lens will illuminate the understanding about the broader societal factors that are the major drivers of drug use among young people [32, 57]. This approach is more likely to inform interventions that address the social conditions of African Canadian adolescents’ daily lives, including experiences of racial discrimination. Also, the findings reveal that adverse health outcomes related to the experiences of racial discrimination were different for boys, girls, immigrant, and Canadian-born African adolescents. Boys in this study experienced greater mental health issues and poorer emotional well-being. This finding is consistent with other studies that have shown that Black males experience greater socioemotional disturbance related to racial discrimination, which also predicts poorer health outcomes in adulthood [3, 5, 59].

Although Canadian-born adolescents reported poor health outcomes, immigrant African adolescents had worse health outcomes, which corroborates poorer health outcomes for immigrants that have been documented in previous studies [52]. Given the selective immigration policies, community support for new immigrants, the healthy immigrant effect has been shown to be protective for most immigrant population. However, the better health among immigrants compared to the larger population tend to wane with longer years of living in the host country [16, 47]. This evidence was supported by the findings of the current study, as most of the African adolescents have lived in Canada for more than six years. Hence, it is not surprising that immigrant African adolescents reported the worst health outcomes.

Furthermore, African Canadians who experienced racial discrimination were less likely to receive mental and emotional health services compared to those who did not report racial discrimination. This finding is consistent with other studies that have found poor health seeking behaviour among individuals with emotional problems, particularly racial and ethnic minorities [13, 61]. Although racial socialization and parental support improve coping and emotional well-being, evidence indicate that many adolescents who experience racial discrimination may not receive the emotional support from their families to deal with the negative mental health issues associated with racial discrimination [31, 42].

The study has some limitations that are worthy to note. As with all secondary analysis, the study was limited to the variables that are available in the data. Also, the survey asked only a single question about racial discrimination, and it is possible that students may have had different perceptions of what counts as racial discrimination. Also, experiences of racial discrimination could be normalized in the everyday life of the adolescents, which may affect reporting of those experiences. Additionally, the survey that was used for the analysis asked a question that required adolescents to identify racial and ethnic background as African. Consequently, the analysis did not evaluate the cultural diversity of Blacks, and the socially constructed identities and racial ethnic categories that might influence experiences of racial discrimination [28]. Hence, intergroup differences in experiences of racial discrimination and link with the health and well-being of African adolescents is a potential area for future study. Furthermore, the survey does not differentiate the contexts where racial discrimination was experienced, nor did the survey ask how often the students experienced discrimination, and from whom (e.g., peers, adults, people known or strangers). More detailed documentation of the frequency, type and context of discrimination would help in developing targeted interventions to prevent or reduce experiences of racial discrimination and the health outcomes. These areas are worth exploring in future studies.

Also, other characteristics that were not measured in the population dataset may influence experiences of racial discrimination, or health behaviours. For example, personality traits, racial socialization, and parental support may affect self concept and self esteem in the adolescents. This might increase or reduce racial discrimination and negative health outcomes. Additionally, socio-economic status of the family might play a role in mitigating or worsening experiences of racial discrimination. Thus, studies are needed to examine individual and family level risk and protective factors that may worsen or mitigate reporting of racial discrimination. Furthermore, gender was measured as a binary variable, which did not address the intersectional experiences of gender diverse and non-conforming adolescents, who experience enhanced racial discrimination and health inequities. Considering that as early as age 10, children are aware and recognize ethnic and skin colour differences, as well as overt and covert actions that are discriminatory, we recommend assessment of experiences of racial discrimination among older children.

Despite these limitations, the strength of the study lies in the use of a population-based survey to provide important insights about the experiences of racial discrimination among African Canadian adolescents in British Columbia. Additionally, the study reveals the differential experiences of racial discrimination by boys, girls, immigrants, and African adolescents who were born in Canada. The link between experiences of racial discrimination and the health and well-being of African Canadian adolescents are useful for providing targeted as well as gender-specific interventions to mitigate the negative health outcomes of experiencing racial discrimination.

## Conclusion

The increasing trends in racial discrimination and the associated adverse health outcomes found in this study are likely to contribute to widening health inequalities for African Canadian adolescents. Health professionals and social workers are well positioned to work with adolescents, families, and communities to create awareness about racial discrimination and the associated health outcomes, provide direct care to adolescents who experience racial discrimination and link them with relevant resources to cope with the negative effects of racial discrimination. These professionals and other relevant stakeholders can play a significant advocacy role informing public health policy about racial discrimination, collaborating with schools, governmental and non-governmental agencies in reducing racial discrimination and the related health issues.

## Data Availability

The datasets used in this study were used with permission from the McCreary Centre Society. Restrictions apply to the access of these data, which are not publicly available. Please contact the McCreary Centre Society for potential access to the data.

## Abbreviations

HSDA: Health Service Delivery Area
BCAHS: British Columbia Adolescent Health Survey
BC: British Columbia
AOR: Age-Adjusted Odds Ratio
OR: Odds Ratio
MCS: McCreary Centre Society
